# Carcinoma Matrix Controls Resistance to Cisplatin through Talin Regulation of NF-kB

**DOI:** 10.1371/journal.pone.0021496

**Published:** 2011-06-24

**Authors:** Karen E. Eberle, Hope A. Sansing, Peter Szaniszlo, Vicente A. Resto, Allison L. Berrier

**Affiliations:** 1 Department of Oral and Craniofacial Biology, School of Dentistry, Louisiana State University Health Sciences Center-New Orleans, New Orleans, Louisiana, United States of America; 2 Department of Otolaryngology, University of Texas Medical Branch Cancer Center, University of Texas Medical Branch Health, Galveston, Texas, United States of America; University of Hong Kong, Hong Kong

## Abstract

Extracellular matrix factors within the tumor microenvironment that control resistance to chemotherapeutics are poorly understood. This study focused on understanding matrix adhesion pathways that control the oral carcinoma response to cisplatin. Our studies revealed that adhesion of HN12 and JHU012 oral carcinomas to carcinoma matrix supported tumor cell proliferation in response to treatment with cisplatin. Proliferation in response to 30 µM cisplatin was not observed in HN12 cells adherent to other purified extracellular matrices such as Matrigel, collagen I, fibronectin or laminin I. Integrin β_1_ was important for adhesion to carcinoma matrix to trigger proliferation after treatment with cisplatin. Disruption of talin expression in HN12 cells adherent to carcinoma matrix increased cisplatin induced proliferation. Pharmacological inhibitors were used to determine signaling events required for talin deficiency to regulate cisplatin induced proliferation. Pharmacological inhibition of NF-kB reduced proliferation of talin-deficient HN12 cells treated with 30 µM cisplatin. Nuclear NF-kB activity was assayed in HN12 cells using a luciferase reporter of NF-kB transcriptional activity. Nuclear NF-kB activity was similar in HN12 cells adherent to carcinoma matrix and collagen I when treated with vehicle DMSO. Following treatment with 30 µM cisplatin, NF-kB activity is maintained in cells adherent to carcinoma matrix whereas NF-kB activity is reduced in collagen I adherent cells. Expression of talin was sufficient to trigger proliferation of HN12 cells adherent to collagen I following treatment with 1 and 30 µM cisplatin. Talin overexpression was sufficient to trigger NF-kB activity following treatment with cisplatin in carcinoma matrix adherent HN12 cells in a process disrupted by FAK siRNA. Thus, adhesions within the carcinoma matrix create a matrix environment in which exposure to cisplatin induces proliferation through the function of integrin β_1_, talin and FAK pathways that regulate NF-kB nuclear activity.

## Introduction

Nearly 80% of stage III and IV oral and tongue cancers are resistant to cisplatin based chemotherapies [1], [2]. In breast, ovarian and lung cancers, the composition of the tumor stroma changes during tumorigenesis. Changes in the tumor stroma include enhanced deposition of matrix proteins such as laminin and collagen, an increase in remodeling of the matrix associated with an increase in fibrillar content and an increase in stromal rigidity or mechanical tension. These changes have been linked to promoting tumor cell progression, motility, invasion and resistance to chemotherapeutic agents [3], [4], [5], [6], [7], [8].

Fibroblasts isolated from the stroma of different staged breast tumors have been utilized to generate tumor-fibroblast matrices [9]. Adhesion of cancer cell lines to these fibroblast matrices is sufficient to confer resistance to chemotherapeutics. However, it is currently not known whether carcinomas secrete a matrix that is sufficient to control chemoresistance. Integrins mediate adhesion to extracellular matrices and in breast cancer cell lines, integrin β_1_ function is required for adhesion to tumor-fibroblast matrices to induce chemoresistance [9], [10], [11]. It is currently not known whether oral carcinoma integrins and integrin downstream signaling pathways control chemoresistance while adherent to the carcinoma matrix [12], [13].

Talin and Src are proteins that associate with integrin cytoplasmic domains in oral carcinomas and function in adhesion-dependent processes [14], [15], [16], [17]. Src downstream signaling regulates survival, apoptosis, spreading, invasion and metastasis [12], [14], [18]. In an embryonic epitheliod cell line, constitutively active Src and integrin β_1_ cooperatively regulate cisplatin chemosensitivity [12]. Knockdown of Src was recently found to inhibit matrigel invasion and proliferation in oral carcinoma cells [17]. In MEFs, Src induces resistance to cisplatin by modulation of connexin 43 function in cell-cell contacts [19]. In ovarian carcinomas with constitutively active Src and FAK, treatment with pharmacological inhibitors of Src reduce survival of cisplatin treated cells [18]. Whether integrin β_1_ and Src cooperatively signal during the oral carcinoma response to cisplatin is poorly understood.

Talin functions in prostate cancer invasion, metastasis and anoikis or cell death induced by detachment from the matrix [20]. Studies in our lab demonstrate that knockdown of talin in oral carcinoma cells inhibits matrigel invasion, disrupts spreading on collagen I and laminin I, reduces proliferation and induces cisplatin chemoresistance [17]. Signaling events elicited by adhesion to the carcinoma matrix that are dependent on talin function in oral carcinomas are poorly understood.

A disruption of talin expression in fibroblasts reduces FAK activation [21] and the overexpression of talin in a prostate tumor cell line activates FAK, Src and Akt signaling [20]. Talin overexpression was recently linked to a high risk for aggressive oral carcinomas [22]. In addition, overexpression of a talin head domain that is defective in integrin activation results in the reduction of Src, FAK and PI-3K signaling in oral carcinomas [22]. Adhesion of breast carcinoma lines to tumor fibroblast-matrices induces similar levels of FAK activation regardless of whether the cell lines are chemosensitive or chemoresistant suggesting that the levels of adhesion induced FAK activity do not correlate with chemoresistance and furthermore, it is possible that FAK-independent pathways regulate chemoresistance in breast carcinomas [9], [11]. The role of FAK in the oral carcinoma response to cisplatin while adherent to carcinoma matrix is poorly understood.

Our current studies examined whether adhesion to the matrix secreted by cisplatin resistant oral carcinoma cells (carcinoma matrix) is sufficient to alter tumor cell behavior following exposure to cisplatin. Our findings demonstrate that adhesion to carcinoma matrix was sufficient to signal for proliferation following treatment with cisplatin. This was observed in two oral carcinoma cell lines, a cisplatin resistant HN12 (IC_50_ 10 µM) [23] and a cisplatin sensitive JHU012 (IC_50_ 1 µM) [24]. We studied the requirement for oral carcinoma integrin β_1_ in the proliferative response to cisplatin of carcinoma matrix adherent cells. We focused on the signaling events that connect matrix adhesion to cisplatin induced proliferation and detected a role for NF-kB signaling in the response to cisplatin. Due to the role of integrins and NF-kB in the matrix adhesion response we examined whether talin regulates both cisplatin induced proliferation and NF-kB signaling. Our studies show that integrin β_1_, talin and FAK regulate NF-kB signaling and cisplatin induced proliferation for oral carcinomas adherent to carcinoma matrix.

## Results

### Adhesion to carcinoma matrix and integrin β_1_ control cisplatin induced proliferation of oral carcinomas

**Figure 1:**
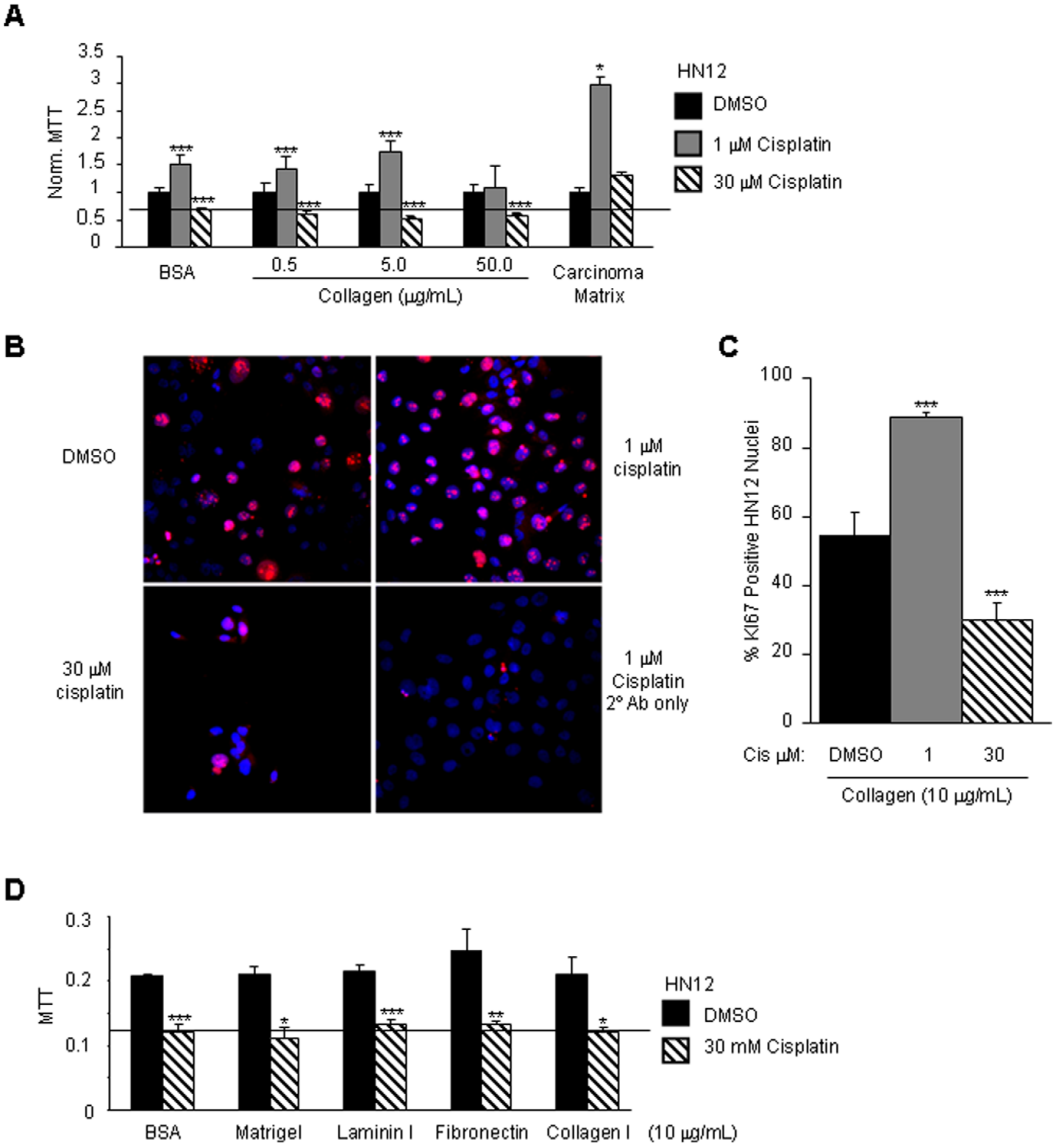
The carcinoma matrix mediates cisplatin induced tumor cell proliferation. The proliferation of HN12 cells adherent to matrix proteins following treatment with vehicle DMSO or cisplatin for 48 hours is shown. A. Proliferation of HN12 cells adherent to BSA (0.1%), collagen I (0.5, 5.0 and 50.0 µg/mL) or carcinoma matrix. Proliferation is normalized to DMSO-treated cells for each substratum. B. Ki67 staining of HN12 cells adherent to collagen and treated with DMSO or cisplatin (1 and 30 µM). Shown are representative fluorescent images of the anti-Ki67 and control secondary antibody staining. The nuclear compartment was visualized by co-staining with DAPI. C. Shown in the bar graph is the mean percentage of HN12 nuclei that are positive for Ki67 in HN12 cells adherent to collagen and treated with vehicle DMSO or cisplatin (1 and 30 µM). Error bars indicate standard deviations. D. Proliferation of HN12 cells adherent to BSA (0.1%), matrigel, laminin I, fibronectin or collagen I (10 µg/mL) and treated with vehicle DMSO or 30 µM cisplatin. Error bars on the mean proliferation are standard deviations. These experiments were performed in three separate trials in quadruplicate. Student's t-test *p* values, *p<0.05, **p<0.01, ***p<0.005.

It was not certain whether adhesion to matrix secreted by oral carcinoma cells is sufficient to alter the tumor cell response to cisplatin. Previous reports indicate that cisplatin at a dose of 10 µM fails to change proliferation of HN12 cells in a conventional culture system with medium containing serum [23]. To study the role of the carcinoma matrix, cisplatin resistant HN12 cells [23] were seeded onto tissue culture dishes coated with carcinoma matrix. Proliferation of replated cells was assayed by MTT and compared in the presence of vehicle DMSO and cisplatin. Proliferation was assayed for cells adherent to carcinoma matrix in serum-free medium (Figure 1A). To monitor whether tumor cell adhesion independent of the carcinoma matrix is sufficient to control the response to cisplatin, cells were replated onto BSA and collagen coated wells. Treatment of the adherent HN12 cells with 1 µM cisplatin induced proliferation on BSA 1.52-fold (*p* = 0.007) and on collagen I 1.73-fold (0.5 and 5 µg collagen respectively, *p* = 0.0046 and 0.0007). Adhesion to collagen I and treatment with cisplatin at 30 µM reduced proliferation 49% compared to DMSO-treated cultures (0.5, 5.0 and 50 µg/ml collagen respectively, *p* = 0.001, 0.0002 and 0.0006). In HN12 cells adherent to carcinoma matrix, treatment with cisplatin at 1 µM increased proliferation 2.97-fold (*p* = 0.01) whereas 30 µM cisplatin induced proliferation 1.32-fold (*p* = 0.07). Thus, adhesion to the carcinoma matrix and treatment with cisplatin induces tumor cell proliferation in serum-free medium.

The ability of 1 µM cisplatin to induce proliferation of HN12 cells adherent to collagen I was confirmed using a second assay for proliferation, we determined the percentage of nuclei positive for the proliferation antigen, Ki67. Fluorescent images of the anti-Ki67 immunofluorescence stained HN12 cells are shown (Figure 1B). The percentage of nuclei positive for Ki67 increased 1.63-fold (*p*<0.005) after treatment with 1 µM cisplatin for 48 hours and treatment with 30 µM cisplatin reduced the percentage of Ki67 positive nuclei by 45% (*p*<0.005) (Figure 1C). The results of the Ki67 nuclear staining reproduced the MTT observations suggesting that adhesion to the carcinoma matrix and treatment with cisplatin indeed induces proliferation of HN12 cells.

The carcinoma matrix contains basement membrane proteins such as collagens, laminins and fibronectin [25], [26]. It seemed likely that adhesion to these purified matrix proteins or Matrigel, a basement membrane extract from EHS tumors [27], would similarly mediate cisplatin induced proliferation. HN12 cells adherent to purified preparations of BSA, Matrigel, laminin I, fibronectin or collagen I were treated with DMSO or 30 µM cisplatin and 48 hours later proliferation was assayed by MTT. Treatment with 30 µM cisplatin reduced proliferation for cells adherent to BSA by 41% (*p* = 0.002), Matrigel 47.5% (*p* = 0.012), laminin I 38.8% (*p* = 0.001), fibronectin 46.9% (*p* = 0.009) and collagen I 48.9% (*p* = 0.012) (Figure 1D). Thus, adhesion to carcinoma matrix and treatment with 30 µM cisplatin induces tumor cell proliferation, a phenotype that is not observed for HN12 cells adherent to a variety of purified matrix proteins or basement membrane extract.

**Figure 2:**
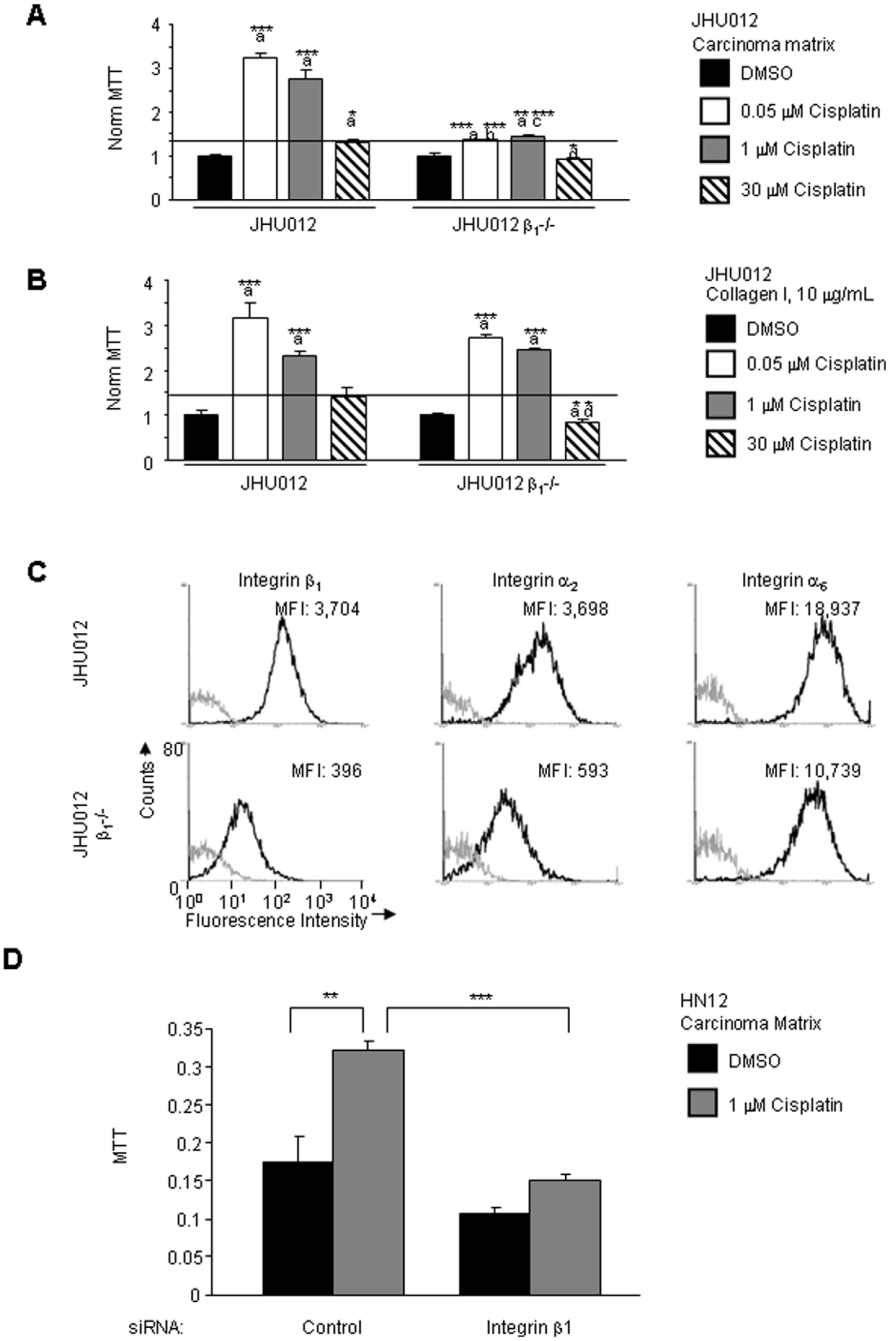
Role of integrin β_1_ in cisplatin induced oral carcinoma proliferation. Proliferation of JHU012 and JHU012 integrin β_1_ deficient cells adherent to A. carcinoma matrix and B. collagen I (10 µg/mL). Cells were treated for 48 hours with DMSO or cisplatin (0.05, 1, 30 µM) as indicated. Shown in the bar graph is the mean proliferation and error bars that indicate standard error. The horizontal line indicates the proliferation of control JHU012 cells after treatment with 30 µM cisplatin. Statistical comparisons for each substratum, a = JHU012/DMSO, b = JHU012/0.05 µM, c = JHU012/1 µM, d = JHU012/30 µM. C. Analysis of surface expression levels of integrin β_1_, α_2_ and α_6_ subunits in JHU012 and JHU012 β_1_ deficient cells by flow cytometry. Shown are overlayed histograms, grey is the isotype control and black is the indicated integrin subunit. MFI: Mean Fluorescence Intensity. D. Proliferation of HN12 cells adherent to carcinoma matrix. Control and integrin β_1_ siRNA transfected cells were treated for 48 hours with DMSO or 1 µM cisplatin as indicated. Shown in the bar graph is mean proliferation and error bars indicate standard error. Student's t-test *p* values, *p<0.05, **p<0.01, ***p<0.005.

It is possible that HN12 cells have an aberrant proliferative response to cisplatin because of their known resistance to cisplatin. To test this possibility, the response to cisplatin was studied in the oral carcinoma cell line JHU012 that is relatively more sensitive to cisplatin sensitive (IC_50_ 1 µM) [24]. JHU012 cells replated on the carcinoma matrix were treated with a wider range of cisplatin concentrations (0.05, 1, to 30 µM) because of the greater cisplatin sensitivity of these cells. Surprisingly, in JHU012 cells the cisplatin response at 0.05 and 1 µM was an induction of proliferation (3.25- and 2.77-fold, respectively *p* = 0.0008 and 0.0046) (Figure 2A). Treatment with 30 µM cisplatin induced proliferation 1.32-fold (*p* = 0.044). As a control, the JHU012 cells were seeded onto collagen I. JHU012 proliferation was induced in response to cisplatin at 0.05 and 1 µM (3.16- and 2.32-fold respectively, *p* = 0.0022 and 0.00018). Treatment with 30 µM cisplatin in the collagen adherent cells failed to reduce JHU012 proliferation below the levels in DMSO treated cells (1.40-fold induction, *p* = 0.107). Adhesion to carcinoma matrix or collagen I and treatment with cisplatin induces proliferation of JHU012 cells.

It was not clear whether integrin β_1_ function is required for cisplatin to induce proliferation of JHU012 cells adherent to carcinoma matrix or collagen. JHU012 cells deficient in integrin β_1_ expression were assayed for the response to cisplatin while adherent to carcinoma matrix (Figure 2A) or collagen (Figure 2B). JHU012 cells deficient in integrin β_1_ attached to the carcinoma matrix and collagen I similar to the parental JHU012 cells (data not shown). Reduction in the surface levels of integrin β_1_ in the integrin β_1_ deficient JHU012 cells was shown by flow cytometry (Figure 2C). The mean fluorescence intensity for surface levels of integrin β_1_ in JHU012 control and integrin β_1_-/- cells is 3704 and 396, respectively, indicating a 9.35-fold reduction in integrin β_1_ expression.

Treatment of integrin β_1_ deficient JHU012 cells with 0.05 or 1 µM cisplatin while adherent to carcinoma matrix induced proliferation 1.36- and 1.45-fold respectively (*p* = 0.003 and 0.013). Parental JHU012 cells induced proliferation 3.25- and 2.77-fold (comparison of JHU012 parental to integrin β_1_ deficient at 0.05 and 1 µM cisplatin, *p* = 0.003 and 0.0098) (Figure 2A). After treatment with 30 µM cisplatin, cell proliferation was reduced by 7% in the integrin β_1_ deficient cells (*p* = 0.14). Thus, if adherent to carcinoma matrix, integrin β_1_ is required for maximal oral carcinoma proliferation in response to 0.05, 1 and 30 µM cisplatin.

Integrin β_1_ deficient JHU012 cells adherent to collagen and treated with either 0.05 or 1 µM cisplatin resulted in similar levels of proliferation (2.71- and 2.46-fold respectively (*p* = 0.0009, 0.0002)) (Figure 2B). Comparison of the proliferation of JHU012 parental and integrin β_1_ deficient lines adherent to collagen after treatment with cisplatin at 0.05 and 1 µM revealed no significant alteration in the levels of proliferation in integrin β_1_ deficient cells (*p* = 0.233 and 0.135). Exposure to 30 µM cisplatin reduced proliferation in the integrin β_1_ deficient JHU012 cells adherent to collagen by 18% (*p* = 0.011). Thus, integrin β_1_ function is required for collagen adherent JHU012 cell proliferation in response to 30 µM cisplatin.

Our data demonstrates that JHU012 cells adherent to carcinoma matrix require integrin β_1_ for cisplatin induced proliferation. In JHU012 cells adherent to collagen I, 30 µM cisplatin induces integrin β_1_ dependent proliferation. In contrast, treatment with a lower dose of cisplatin at 1 µM induces proliferation independent of integrin β_1_. Cisplatin at 1 µM may induce proliferation of collagen adherent cells through either integrin receptor dependent or non-integrin receptor mechanisms. Proteins secreted by the carcinoma during 3 days in culture may engage integrin β receptors in addition to integrin β_1_ and these integrins may promote cisplatin induced proliferation. Alternatively, non-integrin receptor pathways may induce proliferation following treatment with cisplatin such as the EGFR [28].

It was subsequently determined whether integrin β_1_ is important for cisplatin induced proliferation in HN12 cells adherent to carcinoma matrix. HN12 cells adherent to carcinoma matrix with a targeted disruption of integrin β_1_ display a reduction in cisplatin induced proliferation compared to control siRNA transfectants following treatment with 1 µM cisplatin (*p* = 0.0005) (relative proliferation 1 µM cisplatin:DMSO for integrin β_1_ knockdowns 1.41-fold, *p* = 0.0023, for the control siRNA 1.92-fold *p* = 0.012) (Figure 2D). Thus, integrin β_1_ is required for cisplatin to induce maximal proliferation of HN12 and JHU012 cells adherent to carcinoma matrix.

### Intracellular pathways involved in cisplatin induced proliferation

**Figure 3:**
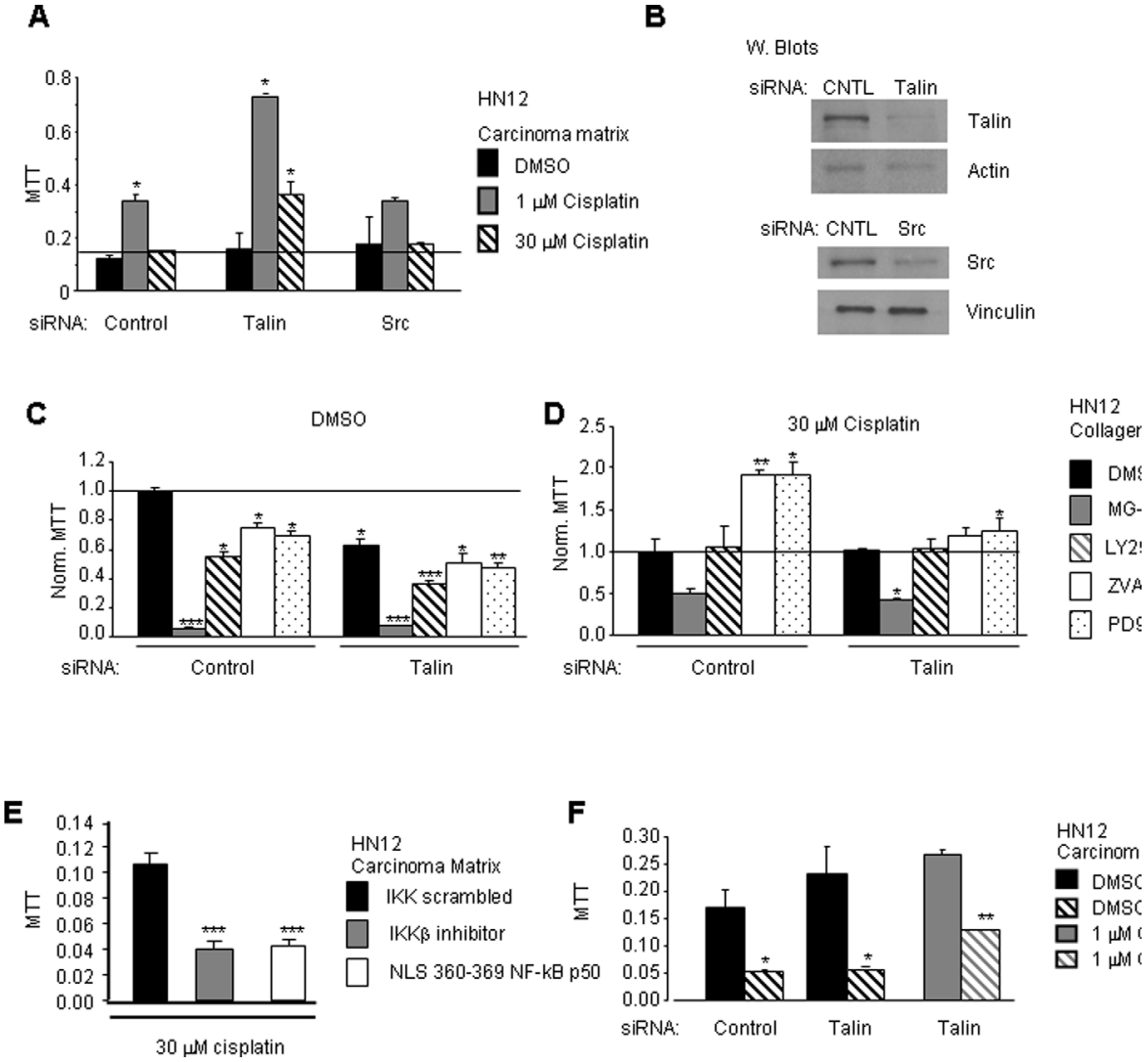
Pharmacological inhibition of oral carcinoma proliferation. A. Proliferation of HN12 cells transfected with talin, Src or control siRNA adherent to carcinoma matrix following treatment with vehicle DMSO or cisplatin (1 and 30 µM) for 48 hours is shown in the bar graph. B. Western blots to analyze the levels of talin and Src in siRNA-treated HN12 cells. C. and D. HN12 cells transfected with talin or control siRNA were assayed for proliferation 48 hours after treatment with C. vehicle DMSO and the indicated inhibitor or D. 30 µM cisplatin and the indicated inhibitor. The mean proliferation with error bars indicating standard error are shown in the bar graph. The data was normalized in C. to control siRNA/DMSO or in D. to control siRNA/30 µM cisplatin. Cells were treated with MG-132 20 µM, LY294002 20 µM, ZVAD-FMK 10 µM and PD98059 20 µM. E. Proliferation of HN12 cells adherent to carcinoma matrix following treatment with 30 µM cisplatin combined with IKK scrambled peptide (67 µM), IKKβ inhibitor (67 µM) or NLS 360–369 NF-kB p50 (100 µg/ml). F. Proliferation of control siRNA transfected HN12 cells adherent to carcinoma matrix following treatment with DMSO alone or DMSO with IKKβ inhibitor (67 µM). Talin siRNA transfected HN12 cells adherent to carcinoma matrix following treatment with DMSO alone or DMSO with IKKβ inhibitor (67 µM), 1 µM cisplatin alone or 1 µM cisplatin with IKKβ inhibitor (67 µM). Student's t-test *p* values *p<0.05, **p<0.01, ***p<0.005. Error bars on the mean proliferation indicate standard deviations. Experiments were performed twice in triplicate.

Due to the known cellular roles of talin and Src and their functions in integrin signaling, talin and Src were likely candidates to control cisplatin induced proliferation mediated by adhesion to the carcinoma matrix [12], [14], [15], [18], [20], [29]. The expectation was talin or Src knockdown would alter integrin signaling and reduce cisplatin induced proliferation. HN12 cells transfected with siRNA-duplexes reduced the steady-state protein levels of talin and Src by approximately 80% (Figure 3B). Control siRNA transfectants and HN12 cells with targeted reductions of talin and Src were replated onto carcinoma matrix and adherent cells incubated with vehicle DMSO or cisplatin (1 and 30 µM) for 48 hours. Proliferation was assayed by MTT (Figure 3A). In control HN12 cells, 1 µM cisplatin increased proliferation 2.96-fold (*p* = 0.001). Proliferation was induced 1.32-fold (*p* = 0.07) after treatment with 30 µM cisplatin. Knockdown of Src induced proliferation 1.88-fold (*p* = 0.1) with 1 µM cisplatin and failed to change proliferation with 30 µM cisplatin. In contrast, knockdown of talin and exposure to 1 µM cisplatin increased proliferation 4.64-fold (*p* = 0.02) relative to the DMSO-treated cells. Treatment with 30 µM cisplatin in the talin knockdowns increased proliferation 2.28-fold (*p* = 0.01) compared to DMSO-treated cells. Furthermore, JHU012 cells adherent to carcinoma matrix with targeted reduction of talin show increased proliferation in comparison to control siRNA transfectants after treatment with 30 µM cisplatin (data not shown). Thus, knockdown of talin increased proliferation induced by cisplatin in HN12 and JHU012 cells adherent to carcinoma matrix.

To further understand the pathways important for proliferation of cisplatin treated cells we tested whether pharmacological inhibitors that target certain signaling pathways will disrupt HN12 proliferation after treatment with cisplatin. Pharmacological inhibitors were selected that target pathways shown by other investigators to regulate chemoresistance in a variety of cellular systems. The selected inhibitors targeted NF-kB (MG-132, a non-specific proteasomal inhibitor) [30] , PI3-K (LY294002) [31], caspases (ZVAD-FMK) [32] and ERK (PD98059) [33], [34].

HN12 cells with siRNA knockdown of talin, and control siRNA transfected cells, were treated with DMSO plus an inhibitor (Figure 3C) or 30 µM cisplatin plus an inhibitor (Figure 3D) and proliferation was assayed 48 hours later. In DMSO-treated cells, the disruption of talin expression reduced proliferation by 37.5% (*p* = 0.018). In the presence of DMSO each of the tested inhibitors similarly altered proliferation in the control and talin knockdowns. MG-132 reduced proliferation in control cells by 94.5% (*p* = 0.0004) and in talin knockdowns 93.1% (*p* = 0.0008). LY294002 reduced proliferation in control cells by 44.6% (*p* = 0.012) and in talin knockdowns 26.5% (*p* = 0.028). ZVAD-FMK or PD98059 reduced proliferation in control cells by 24.2% and 30.0% respectively (*p* = 0.034, 0.011). In the talin knockdowns ZVAD-FMK or PD98059 reduced proliferation 11.6% and 15.8% (*p* = 0.0135, 0.0358). This data suggests that HN12 cell proliferation of talin knockdowns and control cells have similar sensitivity to MG-132 and LY294002.

In the presence of 30 µM cisplatin (Figure 3D) control and talin deficient cells have distinct responses to ZVAD-FMK and PD98059. In control siRNA transfectants, treatment with 30 µM cisplatin and either ZVAD-FMK or PD98059 increased proliferation 1.91-fold (*p* = 0.007 and 0.023) suggesting that inhibition of caspases or ERK pathways leads to enhanced proliferation of cisplatin treated cells. In contrast, ZVAD-FMK with 30 µM cisplatin did not significantly increase proliferation of talin deficient HN12 cells (1.19-fold, *p* = 0.153). PD98059 with 30 µM cisplatin increased proliferation of talin deficient HN12 cells 1.25-fold (*p* = 0.013). Treatment with 30 µM cisplatin and LY294002 did not change the proliferation of control siRNA transfectants (1.06-fold, *p* = 0.44) or talin knockdowns (1.03-fold, *p* = 0.44). Finally, MG-132 with 30 µM cisplatin triggered in control siRNA transfectants a reduction in proliferation by 49.5% (*p* = 0.07) and in talin knockdowns a reduction of 58.2% (*p* = 0.04). Thus, DMSO and cisplatin treated cells differ in sensitivity to LY294002, ZVAD-FMK and PD98059. MG-132 reduced proliferation of DMSO and cisplatin treated cells in control siRNA transfectants and talin deficient cells.

**Figure 4:**
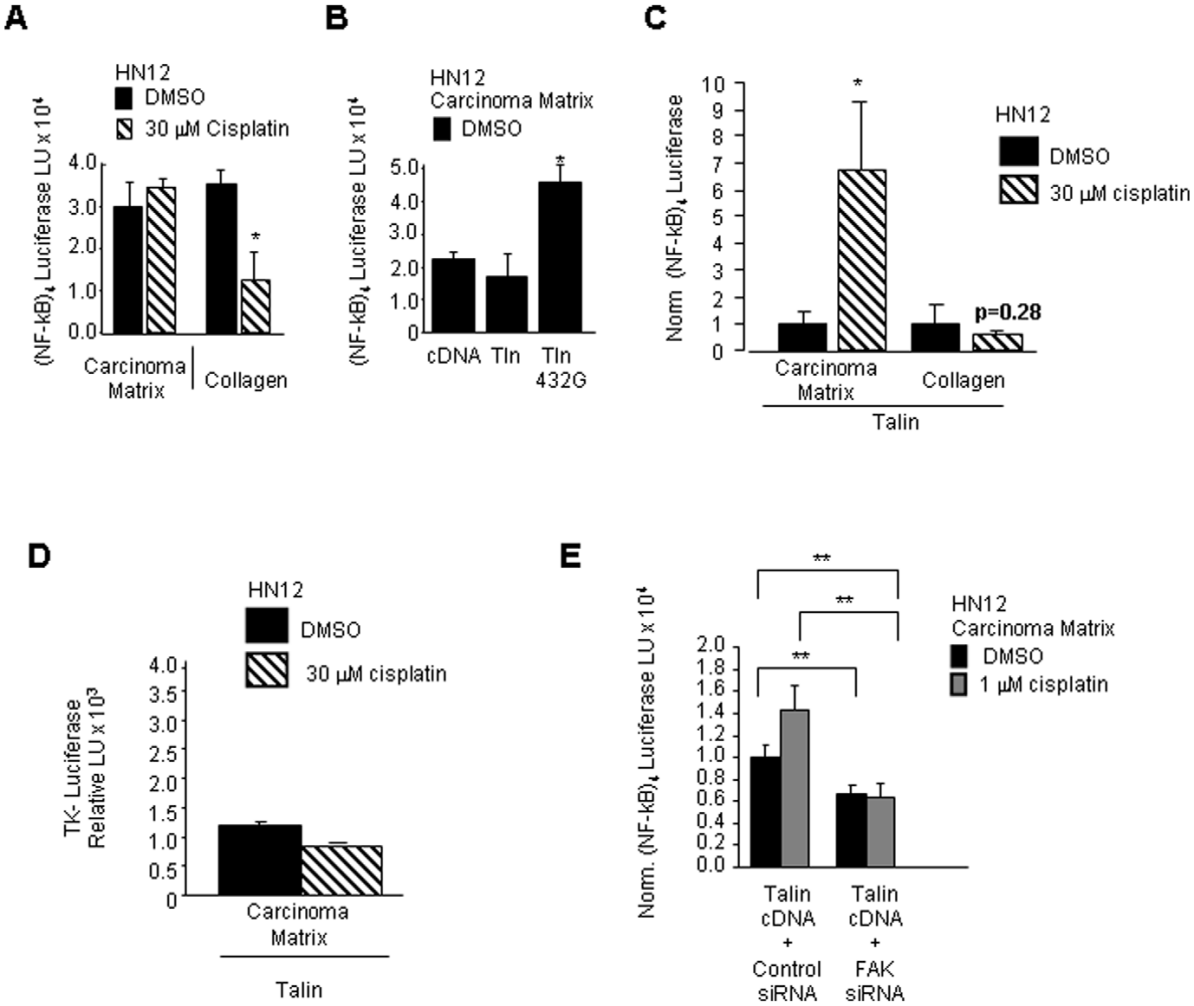
Adhesion to carcinoma matrix and talin overexpression regulate NF-kB activity after treatment with cisplatin. A. HN12 cell transfectants ( (NF-kB)_4_ firefly luciferase together with renilla luciferase) adherent to carcinoma matrix or collagen I were treated for 24 hours with 30 µM cisplatin or vehicle DMSO. Shown in the bar graph is the mean renilla normalized firefly luciferase activity in arbitrary light units with error bars indicating standard deviation. B. HN12 cell transfectants (firefly and renilla luciferase constructs with plasmids containing cDNA expression vectors either cDNA3.1, talin or talin432G) adherent to carcinoma matrix and treated with DMSO for 24 hours. The mean renilla normalized firefly luciferase activity in arbitrary light units with error bars indicating standard deviation is shown in the bar graph. C. HN12 cells transfected with talin cDNA expression vector together with (NF-kB)_4_ firefly luciferase and renilla luciferase were plated onto carcinoma matrix or collagen. Adherent cells were treated with DMSO or 30 µM cisplatin for 24 hrs and renilla normalized firefly luciferase activity is shown in the bar graph with error bars indicating standard error. D. HN12 cells transfected with talin cDNA expression vector together with tk-luciferase (lacking the NF-kB regulatory element) and renilla luciferase adherent to carcinoma matrix were treated with DMSO and 30 µM cisplatin. Renilla normalized firefly luciferase activity is shown in the bar graph with error bars indicating standard error. E. HN12 cells transfected with (NF-kB)_4_ firefly luciferase, renilla luciferase and talin cDNA in combination with either control or FAK siRNA. Carcinoma matrix adherent cells were treated with DMSO or 1 µM cisplatin and renilla normalized firefly luciferase activity is shown in the bar graph with error bars indicating standard error. Student's t-test *p* values *p<0.05, **p<0.01.

To further study the role of NF-kB signaling in the proliferation of HN12 cells adherent to carcinoma matrix we treated adherent cells with 30 µM cisplatin in combination with NF-kB peptide inhibitors. The NF-kB peptide inhibitors included the IKKβ peptide inhibitor (aa T735-E745, blocks NEMO binding with IkB kinase complex) and the NF-kB p50 nuclear localization sequences 360-369 that disrupts p50/p65 nuclear localization. Treatment with either peptide inhibitor reduced proliferation by 60% in comparison to the IKK scrambled peptide control (Figure 3E). We subsequently determined whether disruption of NF-kB signaling affects proliferation of talin deficient HN12 cells adherent to carcinoma matrix. Talin deficient HN12 cells were treated with DMSO or 1 µM cisplatin and the IKKβ peptide inhibitor. The NF-kB inhibitory peptide reduced proliferation of talin deficient cells adherent to carcinoma matrix 4.22-fold and 2.08-fold (*p* = 0.033 and 0.006) respectively in the DMSO and 1 µM cisplatin treated cells (Figure 4D). These results support the notion that proliferation of cisplatin treated HN12 cells adherent to carcinoma matrix may utilize NF-kB signaling.

One possible explanation for talin deficiency promoting proliferation of cisplatin treated cells is talin deficiency may alter the expression levels of regulators in the NF-kB pathway such as increasing the steady state levels of IKKβ or decreasing ikBαexpression. The steady state levels of IKKβ and ikBα in control and talin knockdown HN12 cells were measured by western blot analysis of lysates from HN12 cells adherent to carcinoma matrix prior to and following treatment with cisplatin. There was no detectable change in the steady state expression levels of either IKKβ or ikBα or the levels of serine phosphorylation of IKKβ or ikBα in HN12 cells with reduced expression of talin prior to or following exposure to 30 µM cisplatin (data not shown). Thus, talin deficiency may regulate cisplatin induced proliferation independent of regulating the steady state levels of IKKβ or ikBα ~.

### Talin regulates nuclear NF-kB activity and cisplatin induced proliferation

The phenotype with the pharmacological inhibitors demonstrates that the NF-kB pathway is important for proliferation of cisplatin treated oral carcinoma cells adherent to carcinoma matrix and NF-kB is important for proliferation of talin deficient cells treated with cisplatin. We were interested in whether adhesion to the carcinoma matrix controls NF-kB signaling following treatment with cisplatin. Our expectation was that in cells adherent to carcinoma matrix, NF-kB signaling will increase following treatment with cisplatin. For the collagen adherent HN12 cells we predicted that regulation of NF-kB signaling is independent of cisplatin. Initially, we determined the nuclear NF-kB activity in HN12 cells adherent to carcinoma matrix or collagen I following treatment with DMSO or cisplatin. To test this, HN12 cells were co-transfected with (NF-kB)_4_ Luc, a plasmid containing a multimerized NF-kB regulatory element that controls transcription of a firefly luciferase gene together with a plasmid containing renilla luciferase to normalize for transfection efficiency. Transfectants were replated onto either carcinoma matrix or collagen I and adherent cells were treated with DMSO or cisplatin at 30 µM for 24 hours. Renilla normalized firefly luciferase activity was determined for each condition. Similar levels of NF-kB activity were detected in vehicle DMSO-treated HN12 cells adherent to collagen I or carcinoma matrix. Following treatment with 30 µM cisplatin, NF-kB activity was reduced by 65% (*p* = 0.09) in cells adherent to collagen. NF-kB activity was sustained for cells adherent to carcinoma matrix after treatment with 30 µM cisplatin (1.17-fold increase, *p* = 0.18) (Figure 4A). Thus, adhesion to carcinoma matrix, in contrast to collagen, signals for maintenance of NF-kB signaling in oral carcinoma after exposure to 30 µM cisplatin.

Based on our observation that proliferation increases in talin deficient cells following treatment with cisplatin, we predicted that overexpression of talin should inhibit cisplatin induced proliferation and reduce NF-kB signaling. Since cisplatin induces the activity of calpains and caspases that proteolytically cleave talin, we tested whether overexpression of wild-type and calpain cleavage-resistant talin (talin 432G) [35] are sufficient to inhibit NF-kB activity. In this experiment, HN12 cells were co-transfected with plasmids containing (NF-kB)_4_ firefly luciferase and renilla luciferase in combination with either the empty vector control cDNA3.1, talin cDNA or talin432G cDNA. The transfectants were replated on carcinoma matrix and treated with vehicle DMSO and the levels of renilla normalized firefly luciferase activity are shown (Figure 4B). Similar levels of NF-kB activity were observed in wild-type talin and control cDNA 3.1 transfectants. Expression of talin432G unexpectedly increased nuclear NF-kB activity 2.19-fold (*p* = 0.031). Hence, overexpression of cleavage-resistant talin432G regulates NF-kB activity in oral carcinoma adherent to carcinoma matrix in the absence of cisplatin.

Since adhesion to the carcinoma matrix regulates NF-kB activity following treatment with cisplatin and overexpression of talin can control NF-kB activity it seemed possible that cisplatin treatment of talin overexpressing cells adherent to carcinoma matrix may influence nuclear NF-kB activity. We were interested in determining whether talin overexpression controls NF-kB signaling after exposure to cisplatin in a matrix-specific manner. To test this, HN12 cells were transfected with NF-kB firefly luciferase, renilla luciferase and talin cDNA. Transfected cells were replated onto carcinoma matrix or collagen I and adherent cells were treated with DMSO or cisplatin (30 µM) and the normalized firefly luciferase activity assayed 24 hours later. Unexpectedly, in cells overexpressing talin and adherent to carcinoma matrix, treatment with cisplatin triggered a 6.5 fold induction of NF-kB reporter activity (Figure 4C). In contrast, when the transfectants were plated on collagen, treatment with cisplatin failed to induce NF-kB activity. Thus, overexpression of talin in HN12 cells regulates NF-kB activity following treatment with cisplatin in a carcinoma-matrix specific manner. There was no significant difference in NF-kB levels on collagen for DMSO and cisplatin treated cells indicating that overexpression of talin prevented the cisplatin induced reduction of NF-kB signaling for collagen I adherent cells.

It is possible that talin overexpression in cooperation with cisplatin treatment alters gene expression and may increase basal promoter activity. To test this possibility, talin was overexpressed in HN12 cells and the activity of the minimal TK promoter driving firefly luciferase activity was assayed following treatment with cisplatin and DMSO. This mimimal promoter lacks the upstream multimerized NF-kB cis regulatory element. HN12 cells transfected with talin cDNA, TK-firefly luciferase and renilla luciferase were replated on carcinoma matrix and treated with DMSO or cisplatin (30 µM). Similar levels of normalized firefly luciferase activity were observed (Figure 4D) in DMSO and cisplatin treated cells indicating that cisplatin treatment of talin overexpressing cells does not lead to activation of the minimal TK promoter.

A binding partner of talin currently linked to regulation of cell survival, proliferation and motility is FAK [29], [36], [37]. Whether FAK may be involved in NF-kB signaling induced by cisplatin was not clear because the levels of active FAK were found to be similar in chemosensitive and chemoresistant breast carcinomas [9]. However, FAK activity has been demonstrated to be induced by overexpression of talin [20], [21] and FAK has been shown to regulate NF-kB activity in endothelial cells [38]. We tested whether FAK is required for talin overexpression to induce NF-kB signaling following exposure to cisplatin in oral carcinomas. HN12 cells were transfected with the NF-kB firefly luciferase reporter, the renilla luciferase reporter, talin cDNA and either control or FAK siRNA oligos. The transfectants were replated onto carcinoma matrix and treated with cisplatin (1 µM) or DMSO for 24 hours. The renilla normalized firefly luciferase activity was measured (Figure 4E). The FAK siRNA reduced NF-kB nuclear activity by 56% in the cisplatin treated talin overexpressing cells compared to the control siRNA transfectants. Our data demonstrates that FAK is important for talin to regulate NF-kB activity prior to and following treatment with cisplatin.

**Figure 5:**
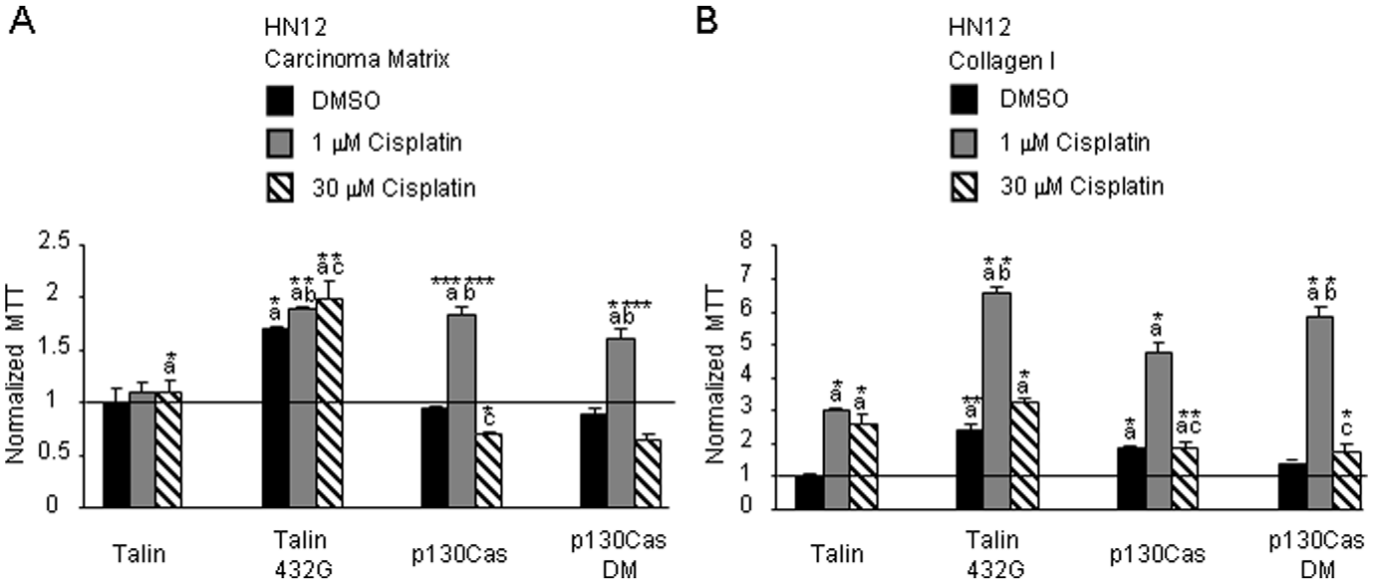
Talin and p130Cas control cisplatin induced proliferation of oral carcinoma. HN12 cell transfectants (plasmids containing cDNA expression vectors either talin, talin432G, p130Cas, p130CasDM (D416E, D748E) and cDNA3.1) were seeded onto A. carcinoma matrix and B. collagen I. Proliferation was measured following treatment with DMSO or cisplatin (1 and 30 µM) for 48 hours. The mean proliferation is shown with error bars indicating standard error. The data was normalized to cDNA 3.1 control transfectants treated with DMSO on each substratum. This experiment was performed 4 times. Statistical comparisons a = control/DMSO, b = control/1 µM, c = control/30 µM. Student's t-test *p* values, *p<0.05, **p<0.01, ***p<0.005.

Since talin overexpression regulates NF-kB activity in cisplatin treated HN12 cells adherent to carcinoma matrix, we subsequently studied whether talin overexpression regulates cisplatin induced proliferation in a matrix-specific manner. For these experiments, HN12 cells were transfected with talin cDNA or talin432G cDNA. Transfected HN12 cells were replated onto collagen or carcinoma matrix and treated with DMSO or cisplatin (1 and 30 µM) and proliferation measured by MTT. In HN12 cells adherent to carcinoma matrix, the proliferation of wild-type talin expressing cells treated with either DMSO or cisplatin (1 or 30 µM) was at levels comparable to control cells treated with DMSO (talin wt: DMSO, 1 and 30 µM cisplatin proliferation respectively of 1.0-, 1.10-, 1.10-fold, *p* values for cisplatin treated cells *p* = 0.1 and 0.041) (Figure 5A). Expression of talin432G increased proliferation 1.70-fold (*p* = 0.025) relative to DMSO-treated control transfectants. This level of HN12 proliferation was maintained in talin432G expressing cells following treatment with cisplatin (1 and 30 µM respectively, 1.89- and 1.99-fold, *p* = 0.020 and 0.043). Thus, expression of talin432G in oral carcinoma adherent to carcinoma matrix increases proliferation. This result is consistent with the observed increase in NF-kB signaling in talin432G expressing cells.

P130Cas is another integrin effector that was preferentially detected in integrin complexes of invasive compared to non-invasive oral carcinomas [17]. In addition, p130Cas is known to regulate survival, motility and apoptosis [39], [40], [41], [42]. It is possible that overexpression of a cleavage resistant p130Cas will similarly influence the ability of the carcinoma matrix to mediate cisplatin induced proliferation. To test this possibility, we examined whether overexpression of p130Cas or cleavage-resistant p130Cas (p130Cas DM) [40] alters cisplatin induced proliferation of HN12 cells adherent to carcinoma matrix. Expression of wild-type and cleavage resistant p130Cas failed to trigger proliferation of HN12 cells (0.95- and 0.89-fold respectively, *p* = 0.37 and 0.32) adherent to carcinoma matrix. Treatment of HN12 cells expressing wild-type or cleavage-resistant p130Cas with 1 µM cisplatin increased HN12 proliferation 1.84- and 1.61- fold relative to DMSO treated control transfectants, (*p* = 0.003 and 0.036) (Figure 5A). Treatment with 30 µM cisplatin decreased proliferation of p130Cas expressing cells by 28% and cleavage-resistant p130Cas expressing cells by 34% (compared to controls/30 µM cisplatin: p130Cas *p* = 0.04, p130Cas DM *p* = 0.067). Thus, expression of cleavage resistant p130Cas does not recapitulate the response to 30 µM cisplatin of talin432G expressing HN12 cells adherent to carcinoma matrix (Figure 5A). Thus, talin 432G and p130CasDM may regulate cisplatin induced proliferation of carcinoma matrix adherent cells through different pathways.

It was not clear whether the overexpression of talin or p130Cas regulates cisplatin induced proliferation in a matrix-specific manner. Adhesion of HN12 cells to collagen and treatment with 1 µM cisplatin induced proliferation whereas 30 µM cisplatin inhibited proliferation. NF-kB activity is reduced by 30 µM cisplatin treatment of collagen adherent cells. Talin overexpression blocked this reduction of NF-kB activity following treatment with 30 µM cisplatin. Hence, it seemed plausible that talin overexpression may alter cisplatin induced proliferation on collagen. Expression of wild-type talin had the same level of proliferation as control mock transfected cells. Expression of talin in collagen adherent cells and treatment with 1 µM cisplatin induced proliferation 3.01-fold (*p* = 0.011). Treatment with 30 µM cisplatin induced proliferation to a similar level as the 1 µM treated cells, 2.59-fold (*p* = 0.034). Expression of talin432G increased proliferation 2.43-fold (*p* = 0.009) relative to control mock transfectants a response that was similar to the carcinoma matrix adherent cells. However, in cells expressing talin432G, treatment with 1 µM cisplatin increased proliferation 6.54-fold (*p* = 0.017). Expression of talin432G and treatment with 30 µM cisplatin increased proliferation 3.26-fold (*p* = 0.032). Thus overexpression of talin significantly increased the proliferation of collagen adherent cells following treatment with cisplatin.

Examination of the role of p130Cas in HN12 cells adherent to collagen revealed that expression of p130Cas increased proliferation 1.84-fold (*p* = 0.05) and p130CasDM increased proliferation 1.36-fold (*p* = 0.109). Following treatment with cisplatin at 1 µM, HN12 cell proliferation increased 4.71-fold and 5.84-fold respectively for wild-type and DM p130Cas (*p* = 0.035, 0.024). Exposure to 30 µM cisplatin induced proliferation 1.84- and 1.77-fold for wild-type and DM p130Cas (*p* = 0.043 and 0.053). The proliferation induced by p130Cas WT and DM at 30 µM cisplatin were lower than the talin wild-type expressing cells (*p* = 0.024, 0.017). Overexpression of either talin or p130Cas induces HN12 cell proliferation after treatment with cisplatin if adherent to collagen I (Figure 5B). Our findings suggest that overexpression of talin regulates both NF-kB nuclear activity and cisplatin induced proliferation.

**Figure 6:**
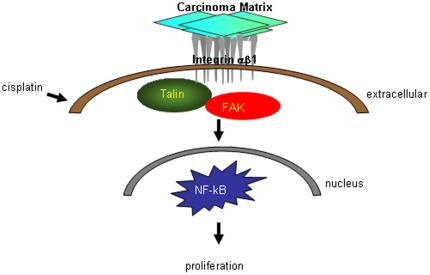
Schematic of oral carcinoma response to cisplatin when adherent to the carcinoma matrix. Cisplatin treatment of oral carcinoma adherent to carcinoma matrix induces proliferation through integrin β_1_ dependent pathways. Treatment with cisplatin regulates NF-kB activity of talin overexpressing oral carcinoma via a FAK-dependent mechanism.

A schematic diagram (Figure 6) contains a summary of the research findings in this study. Oral carcinomas adherent to carcinoma matrix respond to exposure to cisplatin with proliferation in a pathway including integrin β_1_, talin and FAK mediated regulation of NF-kB signaling. Further studies are required to understand the signaling events linking talin/FAK to NF-kB and the matrix components that mediate cisplatin induced proliferation.

## Discussion

Oral carcinoma frequently exhibit resistance to cisplatin based chemotherapy [1]. The mechanisms involved in this resistance are not completely understood. Our studies focused on oral carcinoma chemoresistance to cisplatin and revealed that adhesion to the matrix secreted by oral carcinomas stimulates proliferation following exposure to cisplatin. Integrin β_1_ function is required for adhesion to the carcinoma matrix to induce proliferation in response to cisplatin. Targeted reduction of talin expression in oral carcinoma increased proliferation following exposure to cisplatin. In addition, nuclear NF-kB activity was maintained by adhesion to the carcinoma matrix following treatment with cisplatin. Interestingly, the overexpression of talin in HN12 cells adherent to carcinoma matrix is sufficient to induce nuclear NF-kB activity following exposure to cisplatin. The pathway of talin overexpression regulating cisplatin induced NF-kB signaling is dependent on FAK. Our studies show that carcinoma pathways sensitive to the expression level of talin regulate nuclear NF-kB activity suggesting that the expression levels of talin in oral carcinoma may influence the carcinoma response to cisplatin. In summary, adhesion to carcinoma matrix regulates cisplatin induced proliferation in a pathway involving integrin β_1_, talin and FAK and control of NF-kB signaling.

NF-kB regulates the survival and proliferation of tumor cells following cancer chemotherapy [30], [43]. Genotoxic agents, such as the chemotherapeutic agent cisplatin, modulate the activity of the kinase, IKK, that signals for phosphorylation and degradation of the cytoplasmic NF-kB inhibitor iKBα ~Interestingly, this IKK pathway was previously linked to squamous cell carcinoma progression [44] Degradation of iKBα releases NF-kB from cytoplasmic retention and permits translocation to the nuclear compartment [30]. In talin knockdown HN12 cells we failed to observe changes in iKBα degradation or phosphorylation, or IKKβexpression or phosphorylation prior to or following exposure to cisplatin. Our findings fail to support a model wherein talin regulates the expression level of IKKβ to drive high levels of NF-kB signaling in oral carcinomas following treatment with cisplatin [45], [46].

Other pathways have previously been shown in other cell types to link integrins to regulation of NF-kB. In endothelial cells, integrin receptor signaling activates PKA to antagonize PAK mediated regulation of NF-kB [47]. It is possible that in oral carcinoma cells there is a similar signaling relationship between PKA/PAK and NF-kB and hence, PKA pathways are predicted to disrupt NF-kB activity and oral carcinoma proliferation in response to cisplatin. Future studies will explore this possibility.

Talin overexpression promoted cisplatin induced proliferation of oral carcinoma while adherent to collagen, a matrix that fails to elicit proliferation of the parental HN12 cells in response to 30 µM cisplatin. Perhaps talin overexpression in the oral carcinomas induces activation of FAK and a talin/FAK complex regulates cisplatin induced proliferation. Consistent with this notion, our data shows that FAK siRNA disrupted cisplatin induced NF-kB activation in talin overexpressing oral carcinomas. These studies suggest that FAK inhibitors currently in cancer clinical trials may also function to disrupt oral carcinoma chemoresistance [48], [49], [50]. Future studies will examine this possibility. This involvement of FAK in NF-kB signaling is consistent with previous observations made in endothelial cells during flow [38]. Our findings suggest that the expression levels of talin and potentially other integrin effectors, may modulate the efficacy of genotoxic agents in different microenvironments.

In our studies the targeted disruption of Src failed to alter the cisplatin induced proliferation of oral carcinoma cells. Our findings failed to detect a significant role for Src in oral carcinoma chemosensitivity or chemoresistance. This result contradicts several recent observations. In embryonic epitheliod cells overexpression of constitutively active Src induces chemosensitivity and apoptosis in response to cisplatin [12]. On the other hand, other groups have implicated Src activity in resistance to cisplatin. For example, constitutively active Src in MEFs induces cisplatin resistance through the function of constitutively active Src in regulating phosphorylation of connexin 43 resulting in reduced gap junction function and increased cell survival in response to cisplatin [19]. Also, in ovarian cancer cells with constitutively active Src, treatment with pharmacological inhibitors of Src triggered a reduction in tumor cell survival after cisplatin exposure [18]. In addition, v-Src induced cisplatin resistance by promoting repair of cisplatin-DNA adducts in adenocarcinoma cells [51]. The variable requirements for Src may be attributed to differences in the experimental approaches. Our study focused on Src-deficient oral carcinomas replated on carcinoma matrix in serum-free medium. In the Peterson-Roth and Puigvert studies, overexpression of constitutively active Src^YF^ was found to control the response to cisplatin in serum containing medium for cells replated on tissue culture plastic [12], [19]. These observations suggest that different matrix microenvironments, different cell-types and variable Src expression levels may each contribute to the observed Src-dependent phenotypes following exposure to genotoxic agents.

The carcinoma matrix in an integrin β_1_ dependent manner provides a signal for proliferation in the presence of the genotoxic agent cisplatin. The integrin β_1_ ligand in the matrix that is required for cisplatin induced proliferation is currently not known. Collagen I, fibronectin, laminin I and basement membrane extract each failed to function as a substitute for the carcinoma matrix. In future studies we plan to identify components in the carcinoma matrix that mediate cisplatin induced proliferation.

Our future studies will explore the oral carcinoma pathways that link talin to NF-kB signaling and the induction of proliferation by cisplatin. Further research will study carcinoma matrix components that confer chemoresistance or chemosensitivity. Understanding the intracellular pathways and extracellular matrix factors that coordinate the oral carcinoma response to cisplatin may provide valuable insights into development of agents to improve therapeutic efficacy.

## Materials and Methods

### Cell Lines

HN12 cells are Oral Squamous Cell Carcinoma (OSCC) isolated from the lymph node of a tongue cancer patient [52]. HN12 cells were cultured in DMEM supplemented with fetal bovine serum, penicillin/streptomycin and fungizone in 10% CO_2_ [52]. HN12 cells are p53 deficient and resistant to 10 µM cisplatin in medium containing serum [23].

JHU012 are an OSCC cell line that is sensitive to cisplatin (IC_50_ of 1 µM) [24]. JHU012 β_1_ deficient cells were generated using MISSION TRC shRNA constructs (Sigma) that encode human integrin β_1_ shRNA. Lentivirus was produced in 293FT cells (Invitrogen) using MISSION Lentiviral Packaging Mix (Sigma) [53]. JHU012 cells were infected with lentiviral clones and stably transfected cells were selected in the presence of 1.5 µg/mL puromycin (Cellgro) [54]. Clones were isolated and propagated in RPMI supplemented with fetal bovine serum, penicillin/streptomycin and 3.0 µg/mL puromycin (Invitrogen) and screened for integrin β_1_ knockdown by flow cytometry.

### Calcium Phosphate Transfection

HN12 cells [52] were placed in suspension in antibiotic-free serum-containing DMEM at a density of 1×10^5^ cells/mL. Cells were transfected using calcium phosphate precipitation (Invitrogen). Three pairs of stealth siRNA duplexes were pooled (50 nM total, Invitrogen) for each siRNA transfection [55]. Four days after transfection, cells were harvested for assays and Western blot analysis.

### Coating with Purified Matrix Proteins

Multiwell plates were pre-coated with collagen I, fibronectin, laminin I or Matrigel at 10 µg/mL and blocked with 0.1% BSA in PBS [56].

### Carcinoma Matrix

HN12 cells were cultured at confluency on collagen I (20 µg/mL) coated 24 well plates for at least 12 days in complete DMEM supplemented with ascorbic acid (50 µg/mL). Matrices were denuded of cells as previously described [17], [57], [58].

### MTT Proliferation Assay

HN12 cells were resuspended in serum-free medium then 4×10^4^ cells added to wells in a 24-well plate coated with matrix proteins. Adherent cells were treated with vehicle DMSO (final concentration less than 0.07%) or cisplatin (1 and 30 µM) for 48 hours. Tumor cell proliferation was measured by MTT and data normalized to vehicle DMSO-treated cells. During patient chemotherapy, serum concentrations of cisplatin are approximately 10 µM.

The MTT assay was performed with the reagent (3-(4, 5-dimethylthiazolyl-2)-2, 5-diphenyltetrazolium bromide) (ATCC). The absorbance at 570 nm was measured in a multiwell plate reader (Biotek, synergy2). MTT reagent in the absence of cells was background. Cells adherent to collagen I (10 µg/mL) were treated with pharmacological inhibitors for 48 hours. Cisplatin (EMD chemicals), MG-132, LY294002, ZVAD-FMK and PD98059 (Calbiochem) were resuspended in vehicle DMSO (Sigma). NF-kB pathway inhibitors IKKβ peptide inhibitor, p50 NLS peptide SN50 and IKK scrambled peptide (Biomol) were resuspended in DMSO.

### Immunofluorescence Staining

HN12 cells (3×10^4^) were seeded onto collagen I (10 µg/mL) coated glass coverslips in serum-free medium in 24 well tissue culture plates. The next day, adherent cells were treated with cisplatin (1 and 30 µM) or DMSO. Two days later the cells were fixed (5% sucrose 4% formaldehyde in PBS, pH 7.2), permeabilized with 0.4% Triton X-100 in PBS for 5 minutes, blocked with 3% BSA, 150 mM glycine, pH 7.2 for 30 minutes at room temperature. The blocked cells were stained with Ki67 (mouse, BD Biosciences) and Donkey anti-mouse Cy3-conjugated secondary antibody (Jackson Immunoresearch). Slips were mounted onto glass slides in a drop of prolong gold (Invitrogen) containing DAPI. Fluorescent images were acquired and overlayed using an inverted microscope (Olympus IX81) linked to Slidebook (Intelligent Imaging Innovations) software. The overlayed images were imported into Adobe Photoshop to generate the composite.

### Flow Cytometry

The cell surface expression levels of integrin subunits were determined using direct immunofluorescence staining [59]. Cells were resuspended in FACS buffer (1% fetal bovine serum in PBS, pH 7.2) at a concentration of 1×10^6^ cells/ml (Invitrogen). Phycoerythrin-conjugated anti-integrin antibodies and isotype controls (BD Biosciences) were incubated with the cells for 1 hr on ice. Cells were analyzed using a FACSArray bioanalyzer (BD Biosciences) [60]. Mean fluorescence intensity was determined using WinMDI 2.9 software (The Scripps Research Institute, La Jolla, CA).

### Luciferase Assays

HN12 cells were transfected with plasmids containing (NF-kB)_4_ firefly luciferase (10 µg) and thymidine kinase renilla luciferase (5 µg) (BD Biosciences Clontech), or co-transfected with plasmids (1 µg) containing a eukaryotic expression vector for the following cDNAs talin, talin432G [35], p130Cas or p130CasDM (D416E, D748E) [40], [41]. Two days later, the transfectants were seeded onto either carcinoma matrix or collagen I. Adherent cells were incubated with vehicle DMSO or cisplatin for 24 hours, washed and lysates prepared using passive lysis buffer and the firefly and renilla luciferase activities were measured using luciferase assay reagents (Stop-N-Glo kit, Promega) in a luminometer (Berthold FB12) [61].

### Western Blotting

Whole cell lysates were prepared in 5X SDS sample buffer. Lysates with equivalent levels of actin were tested for the protein levels of the siRNA target. The following antibodies were used talin (Sigma), Src (Upstate Biotechnology), vinculin (Sigma), actin (Sigma) and HRP conjugated secondary antibody (GE Healthcare). The blots were incubated with chemiluminescence ECL reagent (Amersham ECL plus, GE Healthcare) and exposed to Amersham hyperfilm (GE Healthcare) that was developed using a Konica Minolta automatic film developer (Model SRX-101A). The autoradiograms were scanned and images imported into Adobe Photoshop to quantitate the band intensities.
